# The Long-Term Financial and Clinical Impact of an Electronic Health Record on an Academic Ophthalmology Practice

**DOI:** 10.1155/2015/329819

**Published:** 2015-02-24

**Authors:** Michele C. Lim, Roma P. Patel, Victor S. Lee, Patricia D. Weeks, Martha K. Barber, Mitchell R. Watnik

**Affiliations:** ^1^UC Davis Health System Eye Center, UC Davis School of Medicine, 4860 Y Street, Suite 2400, Sacramento, CA 95817, USA; ^2^Department of Statistics and Biostatistics, California State University, East Bay 25800 Carlos Bee Boulevard, Hayward, CA 94542, USA

## Abstract

*Purpose.* To examine financial and clinical work productivity outcomes associated with the use of the electronic health record (EHR).* Methods.* 191,360 billable clinical encounters were analyzed for 12 clinical providers over a 9-year study period during which an EHR was implemented. Main outcome measures were clinical revenues collected per provider and secondary outcomes were charge capture, patient visit coding levels, transcription costs, patient visit volume per provider, digital drawing, and digital imaging volume.* Results.* The difference in inflation adjusted net clinical revenue per provider per year did not change significantly in the period after EHR implementation (mean = $404,198; SD = $17,912) than before (mean = $411,420; SD = $39,366) (*P* = 0.746). Charge capture, the proportion of higher- and lower-level visit codes for new and established patients, and patient visits per provider remained stable. A total savings of $188,951 in transcription costs occurred over a 4-year time period post-EHR implementation. The rate of drawing the ophthalmic exam in the EHR was low (mean = 2.28%; SD = 0.05%) for all providers.* Conclusions.* This study did not show a clear financial gain after EHR implementation in an academic ophthalmology practice. Ophthalmologists do not rely on drawings to document the ophthalmic exam; instead, the ophthalmic exam becomes text-driven in a paperless world.

## 1. Introduction

The Health Information Technology for Economic and Clinical Health (HITECH) Act [1] as part of the American Recovery and Reinvestment Act of 2009 [2] devotes a large sum of public funds towards the digitization of medical records in the hope of reducing the cost of health care and of improving the quality of patient care. It provides a financial incentive to physicians to help offset the cost of implementing an electronic health record (EHR).

Prior to the economic incentives created by the HITECH Act to encourage the “meaningful use” of health information technology, the rate of EHR adoption had been slow in the United States [3, 4]. However, it has gained momentum and the National Center for Health Statistics reported a 78% adoption of EHR among office based physicians in 2013 versus 48% in 2009 when federal incentives became available [5]. The level of EHR adoption amongst ophthalmologists by comparison is low although the rate of rise is similar at 12% in 2008 rising to 32% in 2011 [6].

The slow adoption rate of EHR may be attributed to perceived barriers related to EHR adoption. Some are generalizable to all physicians such as concern about lack of capital resources to invest in EHR despite Federal incentives, loss of productivity during implementation, and insufficient return on investment [3, 6]. In a national survey conducted by the American Academy of Ophthalmology [6], ophthalmologists identified these issues as 3 of the top 4 barriers to implementation. Other barriers are very specific to ophthalmologists such as inadequate tools within the EHR with which to draw the ophthalmic exam [6, 7]. Most ophthalmologists were trained in an era when much of the ophthalmic exam was documented with drawings in paper charts, yet EHR drawing programs are crude and difficult to use and very little data exists as to whether ophthalmologists who have implemented EHRs use them.

We seek to better understand these perceived barriers by measuring the financial and clinical impact of an EHR implementation in an academic ophthalmology practice. We hypothesize that the EHR will support increased financial productivity by improving charge capture and by reducing transcription costs. We expect the use of drawing tools to document the exam in the EHR to be low but that imaging volume will increase in response.

## 2. Methods

This study was a retrospective review approved by the Institutional Review Board of the University of California, Davis Office of Human Research Protection, and it was compliant with the Healthcare Insurance Portability and Accountability Act. All study protocols adhered to the tenets of the Declaration of Helsinki.

The UC Davis Health System Eye Center and its satellite offices at the University of California, Davis Medical Center, implemented an EHR (EpicCare, Epic Systems Corporation, Verona, WI) in October 2007 during the first half of fiscal year 2007-2008. Computerized physician order entry, e-prescribing, messaging, and exam documentation began simultaneously. Previous to this, the medical record was traditionally paper-based and centralized. Information for all primary care and specialty services was kept in a common paper medical record that was delivered to the clinic site for each patient visit.

Financial and clinical work productivity data was analyzed for fiscal years 2002-2003 to 2010-2011. This study period encompassed a five-year period before EHR implementation and a four-year period afterwards. Specifically, clinical revenues, charge capture, visit level codes, number of patient visits per year, and transcription costs were assessed before and after EHR implementation. During the period of study, the number of providers in the department grew from 14 to 23 faculty members and optometrists, 3 to 4 fellows, and 9 to 12 residents. Because of this growth, we chose to analyze our data for a subset of 12 full time providers (faculty ophthalmologists and optometrists) who were in practice a minimum of 3 years before EHR implementation and for 4 years afterwards. House staff was not included in the analysis of revenue and clinical productivity data due to the transient nature of their employment. We were unable to calculate a return on investment for EHR implementation because it was implemented across an integrated academic health center and we could not retrospectively assess specific costs for the UC Davis Eye Center.

### 2.1. Financial and Clinical Productivity

Patient billing summaries were obtained from the UC Davis Eye Center and, from this, clinical revenue data was collected for fiscal years 2002-2003 to 2010-2011. Monetary values were adjusted for inflation based on the Medical Professional Services United States Consumer Price Index for All Urban Consumers using 2011 as the base year. Factors other than the EHR which might affect the rate of payment over time such as payer mix proportion (based on net collections) and reimbursement rate (dollar per work RVU earned) were analyzed before and after EHR implementation.

Charge capture was examined by assessing the change in annual volume of CPT codes billed before and after EHR implementation at each patient visit. Examining the number of CPT codes billed per patient visit per year is an indirect way of assessing whether clinical work performed was captured and billed. The EHR could potentially result in fewer lost documents and promote better capture of procedures performed such as digital imaging, visual fields, and clinic procedures and this should result in a greater number of CPT codes billed after implementation. Ophthalmic procedures or imaging studies performed in the office and that were represented by a unique CPT code were selected for analysis. Procedures that represented evolving or devolving technology or therapies during the study period were eliminated from analysis to avoid confounding of data outcomes. We felt that including these specific imaging procedures would create bias in trying to assess whether EHR implementation was associated with greater charge capture. For example, the use of computer based retina and optic nerve imaging (e.g., optical coherence tomography and confocal scanning laser ophthalmoscopy) and intravitreal injections has risen steadily over the past decade [8] and could falsely elevate the measurement of CPT code billing if included. Likewise, the use of fluorescein and indocyanine green angiography has declined [8] because of the increased use of optical coherence tomography. Therefore, CPT codes for these procedures were eliminated. The CPT code for refraction was eliminated due to a change in coding behavior from within our department during the study period.

Procedures for selected CPT codes that were included were chemo denervation of face muscle (64612), removal of foreign body from the eye (65205, 65210, 65222, 65235, 65260, and 65265), office laser procedures (65855, 66761, and 66821), office slit lamp procedures such as epilation and punctual plug insertion (67820, 67825, 68760, 68761, 68801, and 68840), gonioscopy (92020), visual field testing (92082 and 92083), and imaging procedures (76511, 76512, 76516, 76519, 92136, 92250, 92275, 92285, and 92499) such as ocular ultrasonography, fundus photography, and corneal topography.

Visit level codes were analyzed to assess whether a change in coding levels was evident before and after EHR implementation. Evaluation and Management (E&M) and Eye Codes were divided into new and established higher-level and lower-level visits. New patient higher-level visit codes were defined as 99204-99205 and 92004 and new patient lower-level visit codes were defined as 99201–99203 and 92002. Established patient higher-level visit codes were defined as 99214-99215 and 92014. Established patient lower-level visit codes were defined as 99211–99213 and 92012. The number of patient visits (new and established) per provider FTE (full time equivalent) was collected before and after EHR implementation and this was based on billing data.

Transcription costs were assessed before and after EHR implementation for all providers and data was collected by the UC Davis Health System Health Information Management Services. Prior to EHR implementation, procedure notes and letters to referring physicians and patients were dictated by telephone and transcribed. After EHR implementation, two electronic methods of communicating with referring physicians from outside of the UC Davis Health System were provided. A module within the EHR could create a letter that would then be mailed or the exam and notes could be directly faxed from the EHR to an outside office. Referring providers from within the UC Davis Health System were more likely to receive a message regarding their patients via the EHR messaging system rather than by a formal dictated letter. In addition, transcription costs were averted for procedure notes (injections, lasers, and surgeries) which were originally dictated in a paper environment. After EHR implementation, these items could be created easily with a template within a patient's encounter note in clinic or within an inpatient encounter during a surgery. The choice of using transcription services versus using the EHR to generate communication was left to the discretion of the physician.

### 2.2. Drawing

The rate of digital drawings created within the EHR was assessed. All patient encounters in the EHR over a 4-year period were audited in an automated fashion for the presence of an ophthalmic drawing. The information about documents created in the EHR (document type and time, document description) and the patient visit information (patient identification, date of visit) are stored in an Oracle database. Structured Query Language (SQL) was then used to extract and match visit and drawing information from the database. These results were partitioned by physician level of training (faculty versus house staff), subspecialty, and fiscal quarter. The reports were then analyzed to look for patterns in the rates of digital drawings.

Digital imaging and procedures (e.g., visual fields) volume performed per year were collected to assess for any changes after EHR adoption. Computerized retina and optic nerve imaging procedures (optical coherence tomography (OCT) and confocal scanning laser ophthalmoscopy) were excluded because of the evolution and subsequent increase in use of this relatively new technology [8]. Likewise, fluorescein angiography and indocyanine green angiography were excluded because of the decline in use of these modalities as OCT evolved [8]. We felt that including these specific imaging procedures would create bias in trying to assess whether EHR implementation was associated with a greater volume of imaging and procedures testing.

### 2.3. Statistical Methods

For the models regarding EHR comparisons, mixed effects analysis of variance models were used. Factors in the model were EHR (before/after), “established” (yes or no), and “high-level” (yes or no) as the fixed factors and fiscal year as the random block. EHR nests the fiscal year, and all possible interactions between the fixed effects were included in the model. The analysis was performed using SAS v.9.3 (SAS Institute, Cary, NC). For significant factors, least squares means were used for pairwise comparisons between levels of the interaction terms, for example, the comparison within established patients before and after the implementation of EHR.

## 3. Results

### 3.1. Productivity

**Figure 1:**
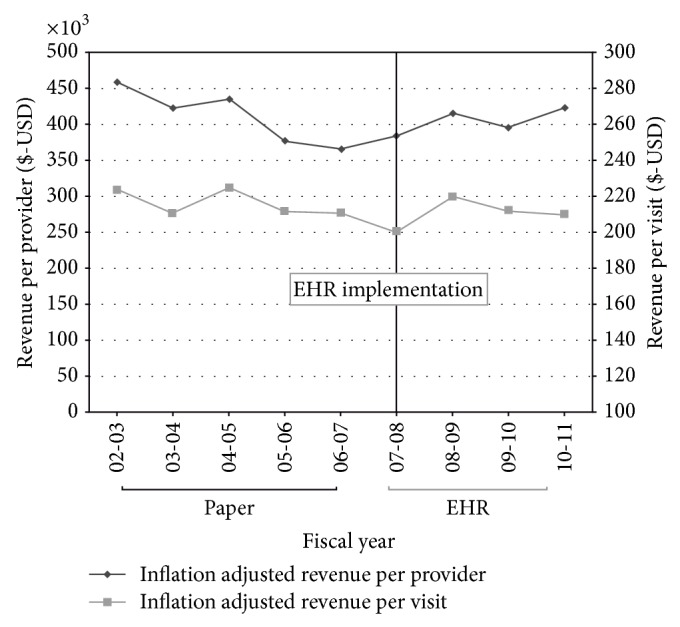
University of California, Davis Eye Center Revenue Data. Inflation adjusted revenues collected per provider and per visit during the period of paper and of EHR documentation. Revenues were adjusted for inflation based on the Medical Professional Services United States Consumer Price Index for All Urban Consumers using 2011 as the base year. USD: United States Dollars.

A total of 191,360 billable clinical encounters were analyzed for 12 clinical providers whose practices were stable throughout the entire study period. The difference in total inflation adjusted clinical revenue collected per provider per year did not change significantly after EHR implementation (mean = $404,198, SD = $17,912) versus before (mean = $411,420, SD = $39,366) (*P* = 0.746) (Figure 1). Likewise, clinical revenue collected per visit per year did not change significantly in the four-year period after EHR implementation (mean = $210.49, SD = $8.00) than in the five-year period before (mean = $215.91, SD = $7.13) (*P* = 0.318) (Figure 1). Elements that could potentially affect clinical revenues such as reimbursement rates for all payer groups (dollar per work RVU) did not change significantly after EHR implementation (mean = $35.35 dollars per RVU, SD = $2.38) versus before ($35.02 dollars per RVU, SD = $0.83) (*P* = 0.277). However, payer mix calculated based on percentage of net revenues collected did show a significant change (*P* < 0.0001) after EHR implementation for the partial risk capitated payer group (14% pre-EHR; 20% post-EHR) and for the self-pay payer group (15% pre-EHR; 11% post-EHR).

Charge capture, defined as the number of selected CPT codes billed per patient visit per year, did not change significantly after EHR implementation (mean = 0.35, SD = 0.03 CPT code billed/patient) versus before (mean = 0.32, SD = 0.03 CPT billed/patient) (*P* = 0.172).

**Figure 2:**
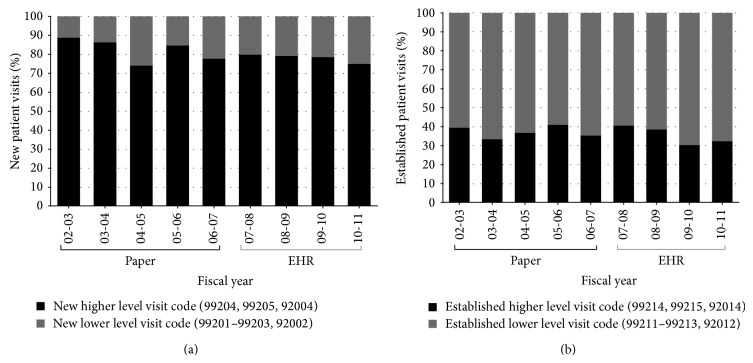
(a) Proportion of new patient and (b) established patient higher and lower level visit codes during the period of paper and of EHR documentation.

The proportion of higher-level visit codes for new patients was 78% (SD = 0.02%) of all visits billed after EHR implementation versus 82% (SD = 0.06%) of all visits billed before (*P* = 0.107). The proportion of higher-level visit codes for established patients was 35% (SD = 0.05%) of all visits billed after EHR implementation versus 37% (SD = 0.03%) of all visits billed before (*P* = 0.404) (Figures 2(a) and 2(b)).

**Figure 3:**
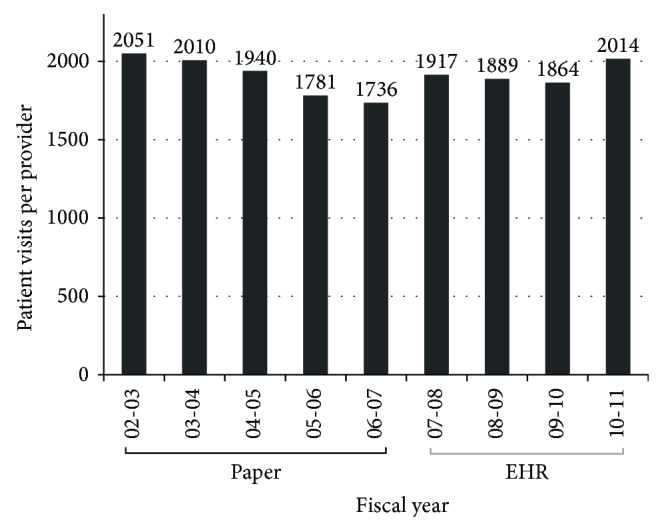
The number of patient visits per provider during the period of paper and of EHR documentation.

Patient volume after EHR implementation (mean = 1920, SD = 65 patients per provider per year) versus before (mean = 1903, SD = 139 patients per provider per year) did not change significantly (*P* = 0.426) (Figure 3).

After EHR implementation, transcription costs averaged $20,768.47 per year (SD = $11,715.64) versus $52,136.87 per year (SD = $12,237.42) before (*P* = 0.01). Transcription costs reached a high of $68,006.24 the year before EHR implementation and using this as a base value, a total savings of $188,951 was realized over the ensuing four year time period of EHR use.

### 3.2. Drawings

**Figure 4:**
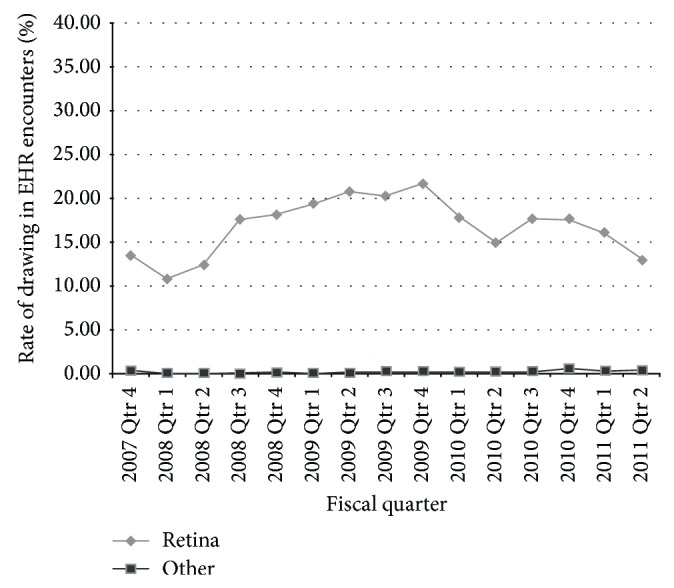
The rate of drawings created in all patient EHR visit encounters over a 4-year period for retina specialists versus other ophthalmologists (comprehensive, cornea, and glaucoma specialists, pediatric ophthalmology, oculoplastics, neuroophthalmology, and residents).

An automated audit of 121,618 total patient encounters over a 4-year period revealed that the rate of drawings made in EHR encounters was low (mean = 2.28%; SD = 0.06%) (Figure 4). The retina subspecialists had higher rates of drawing (mean = 16.78%, SD = 0.03%) for all EHR encounters during this time period in comparison to other ophthalmologists (comprehensive, cornea and glaucoma specialists, pediatric ophthalmology, oculoplastics, neuroophthalmology, and residents (mean = 0.21%, SD = 0.48%)). The number of imaging and diagnostic procedures (automated perimetry, electroretinography) performed per visit did not change significantly after EHR implementation (mean = 0.27, SD = 0.03 images per visit) versus before (mean = 0.25, SD = 0.03 images per visit) (*P* = 0.314).

## 4. Discussion

### 4.1. Financial and Clinical Productivity

Surveys of ophthalmology and nonophthalmology providers identify cost of EHR implementation, questionable return on investment, and loss of productivity as significant barriers to adoption of health information technology [3, 7]. Our findings show that clinical work productivity based on revenue collection, charge capture, and patient visits per provider did not change in the post-EHR era. Though these latter metrics did not increase after EHR adoption, they at least did not decrease, as many ophthalmologists feared would happen. Chiang et al. [9] recently reported financial and productivity outcomes after EHR implementation for a large academic ophthalmology practice. In this study, financial outcomes were expressed as work RVU's generated specifically from patient visit CPT codes and they did not find a significant difference after EHR implementation. The study did note an initial drop in patient volume in the first year after EHR adoption but a trend toward increasing volume occurred in the following 2 years. Past studies of the financial impact of EHR implementation in other areas of medicine have been mixed [10]. A study of 49 community practices representing both primary care and specialty clinics found a mean negative return on investment of $43,743 after EHR implementation [11]. Of the 27 percent of practices that achieved a positive return on investment, several factors were identified that set them apart from practices that reported a loss. They were the ability to increase number of patients seen per day and the ability to increase revenue through higher level coding and fewer rejected billing claims.

We noted changes in payer mix for two groups; the proportion of revenues collected from the partial risk capitated payer group rose while those for the self-pay payer group declined post-EHR adoption. In order to understand how this might affect overall practice revenue collections, the dollar per RVU collected was calculated for each payer group. The self-pay payer group had a higher dollar per RVU (mean = 52.37, SD = 11.11) collected in comparison to the partial risk capitated payer group (mean = 26.10, SD = 0.41) and it is therefore possible that the payer mix changes affected clinical revenues by decreasing collections in the post-EHR era. We did not find an increase in selected procedural charge capture in our practice. We expected to find improved charge capture because in the EHR, documentation is never lost and it is legible. Because the cost of implementing an EHR has been shown to be high in other studies [7, 10, 12], the lack of productivity gains in our department suggests that our return on investment for EHR implementation is likely negative.

Our study did not find an increase in the rate of billing of upper level visit codes for either new or established patients. This is in contrast to other studies that have shown an increase in coding level [9] and some attribute it to better documentation via the use of templates and also to an automatic E/M calculator that recommends the proper code based on the documentation during the visit [13–15]. Recently, “up coding” of visit levels has been noted nationwide and it has received much attention from the Office of Inspector General (OIG) [16], a part of the Department of Health and Human Services. A report, analyzing coding behavior amongst physicians in the United States between 2001 and 2010, found that the percent of the two upper level outpatient visit codes rose by 17% during this period of time [16]. In response to this trend, the OIG will begin to review E and M services “to identify electronic health records (EHR) documentation practices associated with potentially improper payments” [17].

Four years after EHR implementation, transcription costs were reduced by approximately 85% for a total savings of $188,951 during this time period. The new avenues of communication with referring providers and of procedure note creation within the EHR were the presumed explanation for transcription savings. Studies in other areas of medicine show a similar cost savings effect with transcription reduction [18–20]. Barlow et al. [18] studied the economic effect of EHR implementation on a 59-provider multispecialty practice in central Utah. One year after EHR implementation, transcription costs were reduced by 35% for a total savings of $380,000. Transcription savings for specialty groups have also been reported. Patil et al. [20] reported a decrease in transcription costs from $57,962 the year prior to EHR implementation to $7,086 three years later for an eleven provider urology group in an academic center. Though we found a significant savings in reduced transcription costs, other factors such as the cost of hiring information technology staff to implement and support the EHR, physician time spent on documentation by EHR rather than by transcription, and maintenance of the hardware and software costs of the EHR may influence the final return on investment. Nonetheless, several studies that were able to account for these variables have shown a positive return on investment [10, 12, 20, 21] after EHR adoption.

### 4.2. Rates of Drawing

Ophthalmologists voice great concern regarding the ability to draw within an EHR [7, 22]. Our results showed that the rate of drawing in the EHR was low, especially among the anterior segment specialties. The retina specialists are more heavily dependent on a pictorial exam but even so, the rate of drawings created in the EHR for this subspecialty was 20% or less for all encounters in a 4-year period. The majority of the ophthalmic exam was documented with textual descriptions typed on a computer keyboard. Boland et al. [6] showed that a large proportion of survey respondents identified the ability to draw in an EHR as a moderate to significant barrier whether they had or had not implemented an EHR. In a study comparing ophthalmology notes created in an EHR versus on paper, Sanders et al. [23] reported that zero of 150 EHR notes contained a drawing and relied more heavily on text-based descriptions of clinical findings. What is apparent from ours and other's studies is that the mental concept of the ophthalmic exam becomes text-driven in the EHR rather than pictorial, presumably because EHR drawing programs are still crude and difficult to use. Chiang et al. [22] described the frustration that ophthalmologists face when trying to draw in current EHRs and noted that vendors need to develop better methods of incorporating picture-based findings whether they are drawings, annotated templates, or digital images. But before ophthalmologists can adequately guide vendors in their design of future EHRs, they must answer the important question of whether drawings of the ophthalmic exam provide a benefit to clinical interpretation and continuing management of a patient. No studies exist at this point that answer this question.

Because of the poor ability to draw in EHR, we expected the volume of digital imaging to increase after EHR adoption. However, our data did not show this trend. It is possible that the providers were satisfied with their textual description of the eye exam and/or that the added workflow required in sending a patient for additional imaging precluded this behavior.

### 4.3. Limitations

The limitations of this study are that they were performed at a single academic institution within an integrated healthcare system. Thus, the findings may not be generalized to other types of ophthalmology practices such as solo or small groups. In addition, the cost of EHR implementation such as salary support for IT specialists and hardware and software expenses was not recorded on a specialty level. Likewise, potential areas of costs savings such as potential reduction in medical records personnel or cost reduction in the use of paper was not recorded for the UC Davis Eye Center. Therefore, a return on investment could not be calculated. The study period preceded payments from “meaningful use” incentives and, therefore, the impact of this federal aid on financial returns was not applicable. In addition, our conclusion that imaging volume did not significantly rise after EHR implementation may be limited by the fact that we had to eliminate imaging procedures that evolved over the study period such as OCT and confocal scanning laser ophthalmoscopy.

## 5. Conclusion

This study did not show a clear financial gain after EHR implementation in an academic ophthalmology practice. Some benefit in clinical work productivity such as a marked savings via reduced transcription existed but other measures of productivity including clinical revenues, charge capture, visit coding levels, and patient visit volume did not increase after EHR implementation. Lessons learned from this study are that the majority of financial and clinical work productivity metrics did not increase after implementation of an EHR in our academic ophthalmology practice. Another lesson learned is that ophthalmologists who have traditionally documented the ophthalmic exam with drawings now primarily record their clinical findings with text-based descriptions. In effect, the mental concept of the eye exam becomes text-driven not pictorial.
